# Laparoscopic and Robot-Assisted Surgery for Gestational Trophoblastic Disease: A Scoping Review of Patient Selection and Intraoperative Precautions

**DOI:** 10.7759/cureus.117071

**Published:** 2026-09-28

**Authors:** Rei Gou, Makiko Yuda, Hiroko Fukasawa

**Affiliations:** 1 Department of Obstetrics and Gynecology, Yamanashi Red Cross Hospital, Kawaguchiko, JPN

**Keywords:** gestational trophoblastic disease, intraoperative reporting, laparoscopy, morcellation, peritoneal washing, robotic surgery, scoping review, specimen retrieval, uterine manipulator

## Abstract

Guidelines for gestational trophoblastic disease (GTD) state when hysterectomy is indicated, but say little about the operative route or what should be recorded during the operation. We conducted a Joanna Briggs Institute (JBI) scoping review, reported to the Preferred Reporting Items for Systematic Reviews and Meta-Analyses extension for Scoping Reviews (PRISMA-ScR), of laparoscopic and robot-assisted operations that resected or biopsied GTD tissue to establish which patients undergo these operations and what intraoperative precautions the reports contain. PubMed (August 18, 2026; 449 records) and Ichushi-Web (August 20, 2026; 329 records) were searched without language or date restriction, and backward citation chasing added two reports. The review maps what is reported; it estimates neither safety nor effectiveness, and no meta-analysis was performed. In total, 64 original reports describing 67 operations entered the main analysis, alongside 24 Ichushi-Web conference records as a supplementary layer; 54 were case reports, and the literature spans 1995 to 2026. GTD had been diagnosed before 38 of the 67 operations; before the other 29, it had not. The details needed to study intraperitoneal containment were rarely described: use or non-use of a uterine manipulator in five of 36 evaluable reports of operations on a retained uterus, morcellation in three of 36, a retrieval bag in two of 50, peritoneal washing cytology in six of 53, and pneumoperitoneum pressure in none of 52. A mechanical containment token was present in 17 of 57 evaluable reports, but of the 16 that could also be assessed for wording, 12 never used the words spillage, seeding, dissemination, or port-site. Port-site events were charted as an outcome and were not reported in 43 of the 49 reports in which that field was evaluable. Laparoscopic and robot-assisted surgery for GTD has been reported since 1995, mainly as single cases and in a preoperatively mixed population, and the intraoperative detail that would allow containment to be studied is usually absent. The gap this review identifies is one of reporting rather than of technique.

## Introduction and background

Gestational trophoblastic disease (GTD) is a group of rare tumors and tumor-like lesions that grow from the placental tissue of a pregnancy. Most patients are treated without an operation: hydatidiform moles are removed by uterine evacuation, and the malignant forms usually respond to chemotherapy. GTD includes premalignant entities such as complete and partial hydatidiform moles, as well as malignant variants collectively referred to as gestational trophoblastic neoplasia (GTN). The malignant forms include invasive mole, choriocarcinoma, placental site trophoblastic tumor (PSTT), and epithelioid trophoblastic tumor (ETT) [1]. According to the International Federation of Gynecology and Obstetrics (FIGO) risk scoring system, GTN is divided into low-risk and high-risk groups for treatment stratification: low-risk patients receive single-agent chemotherapy, whereas high-risk patients are treated with multi-agent regimens [2-5]. PSTT and ETT behave differently and are relatively chemoresistant; in them, a long interval since the antecedent pregnancy and advanced anatomical stage are more informative prognostically than the FIGO risk score [2]. Reported survival is close to 100% in low-risk and about 90% in high-risk GTN [2]. Surgery is therefore uncommon, but heterogeneous in purpose: histological diagnosis, control of hemorrhage, and resection of chemoresistant residual disease [2,6]. Where disease is confined to the uterus, hysterectomy may itself be curative and is available to patients not wishing to preserve fertility [4,6]. In PSTT and ETT, it is primary rather than salvage therapy, and advanced disease often requires surgery combined with systemic treatment [2]. How often such operations are performed, and using which technique, remains largely undocumented. That is an argument for describing current practice, not for awaiting a trial this incidence cannot support.

Guidelines specify when hysterectomy is indicated, but say less about how it should be performed or what should be recorded intraoperatively. The 2013 European Society for Medical Oncology (ESMO) guideline covers indications and staging but not surgical route [3]. Society of Obstetricians and Gynaecologists of Canada (SOGC) Guideline No. 408, the Spanish Society of Medical Oncology (SEOM) guideline, and the National Comprehensive Cancer Network (NCCN) guidelines all list hysterectomy as a treatment option without specifying whether it should be performed laparoscopically or by laparotomy [4-6]. Only the European Organisation for the Treatment of Trophoblastic Diseases (EOTTD) practical guideline and the subsequent EOTTD-European Society of Gynaecological Oncology (ESGO)-Gynecologic Cancer InterGroup (GCIG)-International Society for the Study of Trophoblastic Diseases (ISSTD) consensus address route directly, and each does so in a single conditional clause: in early PSTT/ETT, “a laparoscopic approach might be feasible but morcellation should be avoided” [7], and, in the 2025 consensus flow diagram, “laparoscopic approach in selected cases if spill-free” [8]. Across these guidelines, the only intraoperative concern named is spillage and the only maneuver named is morcellation; uterine manipulation, specimen retrieval, peritoneal washings, and pneumoperitoneum are not mentioned.

GTD is the major gynecological tumor type whose laparoscopic literature has not been mapped for patient selection or for the intraoperative details needed to study containment. The field has not yet written down what it is doing. Existing syntheses cannot fill that gap. Systematic reviews of whether hysterectomy improves outcome in GTN have, by design, excluded case reports and analyzed only cohort or comparative studies, so the material that carries almost all of the laparoscopic signal falls outside them [9-11]. A scoping review is therefore the design that fits. We ask for which patients, and with what described intraoperative precautions, laparoscopic and robot-assisted surgery is being performed for GTD, including operations in which GTD was not the preoperative diagnosis, and including tumor-like lesions that cannot be distinguished from PSTT before the specimen is examined. We map what is reported and what is not. We do not attempt to establish whether laparoscopy is safe in GTD, and we do not recommend a technique.

## Review

Methodology

Design and Registration

This scoping review follows the Joanna Briggs Institute (JBI) Manual for Evidence Synthesis (scoping reviews chapter) [12] and is reported according to the Preferred Reporting Items for Systematic Reviews and Meta-Analyses extension for Scoping Reviews (PRISMA-ScR) [13]. It maps what laparoscopic and robot-assisted reports of GTD describe. It does not estimate safety or effectiveness and does not include a meta-analysis.

The protocol was registered retrospectively on the Open Science Framework (OSF) (https://doi.org/10.17605/OSF.IO/J3RKU; August 21, 2026) [14]. At registration, the PubMed string (449 records), a nine-report pilot extraction, and the Ichushi-Web search were already in hand. Title and abstract screening of the PubMed yield was finished on August 23, 2026, as completion of that registered search, not as a second search. The registration’s claim that all PubMed-derived records had already been screened on that date is incorrect and is logged as a deviation; the OSF snapshot is not rewritten. No member of the review team authored an included report.

Questions

Primary question: For which patients, and with what described intraoperative technical precautions, is laparoscopic (including robot-assisted) surgery performed for GTD?

Secondary questions: Reported patient characteristics; whether GTD was diagnosed before surgery (axis 1a versus 1b, and within 1b whether oncological sampling was done); description of techniques that can contain spillage or hemorrhage; and reporting of spillage, port-site events, and outcomes. We also charted the journal domain, assigning each report to gynecologic oncology, general obstetrics and gynecology, or a non-gynecological field based on the journal title. It is described in the Results section, but its denominators are too small to support a question, so none is posed.

One expectation was recorded before main extraction, after a nine-report pilot: that manipulator use or non-use would be unreported in most operations on a retained uterus. It is therefore not presented as an untested prediction. The review tests no hypotheses.

Eligibility Criteria

Eligibility followed a population-concept-context (PCC) framework. Population: complete or partial hydatidiform mole, invasive mole, gestational choriocarcinoma, PSTT, ETT, and tumor-like lesions (exaggerated placental site, placental site nodule). Tumor-like lesions were included because they cannot be distinguished from PSTT before surgery and are classified within GTD [1]; a sensitivity analysis excluding them was planned. Eligibility used final histology, not the working diagnosis. Concept: laparoscopic or robot-assisted surgery that resected or biopsied GTD (primary, metastatic, or recurrent), judged at report level. An operation in a patient with GTD that did not reach GTD tissue (for example, cholecystectomy, or diagnostic laparoscopy with later open resection) did not meet Concept. The review is therefore about operations that handle GTD tissue, which is where the guideline conditions on morcellation and spillage apply; purely diagnostic laparoscopy raises pneumoperitoneum and port-site questions but not those conditions. Context: all years, languages, countries, and settings.

The following six reasons were applied in order. If a record met more than one reason, we counted it under the first reason only, so no record was counted twice. Reasons 1 to 3 are sections 5.1 to 5.3 of our registered protocol [14], applied in that order before Concept and design. (1) Residual trophoblast after surgery for ectopic pregnancy (§5.1): histologically normal villus, which is not GTD. GTD arising at an ectopic implantation site remained eligible. (2) Non-gestational origin (§5.2): ovarian and extragonadal tumors were accepted as gestational only where a paternal genome had been demonstrated. Records without that demonstration were excluded as origin-indeterminate. (3) Final histology not a GTD entity, as defined under Population (§5.3). (4) Concept not met, as defined above. (5) Design: reviews, editorials, and pictorial essays, which were kept as sources for citation chasing. Conference records and case reports remained eligible. (6) Concept not confirmable from the abstract when the full text was not obtained. Such reports were excluded rather than retained with imputed values.

One uterine choriocarcinoma was excluded on the Population definition rather than under §5.2, the patient having no history of sexual activity or pregnancy. Adjacent reports of intraperitoneal trophoblast after ectopic pregnancy surgery were kept for the Discussion only; they are not GTD and were not placed in the PRISMA-ScR “excluded at screening” box.

Information Sources and Search

Ichushi-Web was searched on August 20, 2026 (329 records; no year filter; conference records included) and PubMed on August 18, 2026 (449 records; no filters). Executed strings are presented in Appendix A and the registered file search-strategy.txt [14]. A dated re-run of the registered PubMed string on August 23, 2026 (1800:2026/08/18[edat]) recovered 448 records (one registered hit not retrieved; treated as an indexing change). Identifiers from an earlier export that were not in that dated yield were screened and excluded; they are not counted among the registered 449 (Figure 1). The string was not re-run without a date cap.

**Figure 1:**
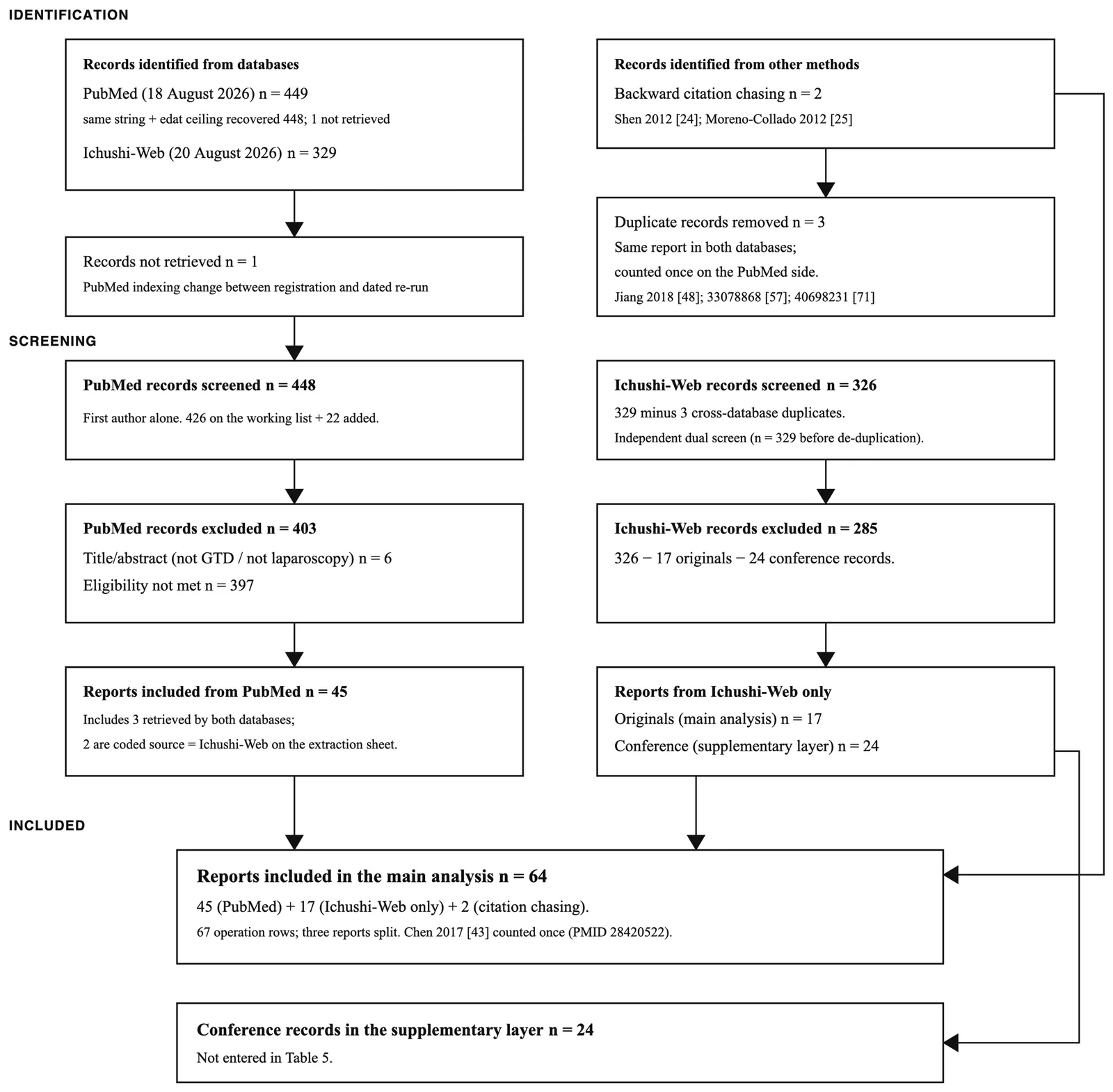
PRISMA-ScR flow of reports. PubMed was searched on August 18, 2026 (n = 449). A dated re-run recovered 448; one registered hit was not retrieved (indexing change). A 522-row working export held 96 extra PMIDs (all excluded); they are not identified records. The 79/420 split belongs to the 544-row working log and is not this figure. Ichushi-Web was searched on August 20, 2026 (n = 329) and dual-screened. Three reports were in both databases and counted once on the PubMed side. Two of those three are coded as Ichushi-Web on the extraction sheet. Backward citation chasing identified two more studies, which the strings did not retrieve. Four further records that the extraction sheet tags as “citation-chased” were in fact retrieved by the registered PubMed string and are counted among the 45 reports included from PubMed rather than in the citation-chasing box. Dual-screen tagging found 18 Ichushi originals; two (Ichushi 1984122591, 1986122913) were excluded at full text (inspection only), leaving 17 Ichushi-only originals. Conference records (n = 24) are a supplementary layer. Adjacent residual-villus reports (Discussion only; not GTD) are not shown as screening exclusions. Eligibility exclusions on PubMed used protocol sections 5.1-5.3, Concept, and design, in that order. The diagram was drawn with a plotting script; no generative artificial intelligence tool was used to create or modify the image. PRISMA-ScR: Preferred Reporting Items for Systematic Reviews and Meta-Analyses extension for Scoping Reviews; GTD: gestational trophoblastic disease; PMID: PubMed identifier

Embase, the Cochrane Central Register of Controlled Trials (CENTRAL), China National Knowledge Infrastructure (CNKI), and Wanfang were not searched. The gap is material for CNKI and Wanfang. Chinese-language reports indexed in PubMed were read in the original language. Gynecological Surgery and Gynaecological Endoscopy are not in PubMed and have carried eligible reports. No language restriction was applied; English, Japanese, and Chinese reports were read in the original language by the first author. Trial registries, theses, and preprint servers were not searched. Authors were not contacted.

Backward citation chasing used the reference lists of narrative reviews retrieved by the searches, of the adjacent residual-villus reports, and of included reports read during extraction. After the main analysis was closed, we searched electronically both the reference lists and the citing papers of the 47 included reports that carry a PubMed identifier (PMID) or a digital object identifier (DOI). We also read the reference lists of the three Ichushi-Web-only reports that have an open-access full text. A candidate was kept only if its title or journal contained both a laparoscopy word and a GTD word. No further eligible report was found. Thirteen Ichushi-Web-only reports and one abstract-only report could not be searched this way, so the reference lists of 14 of the 64 included reports have not been read. Citation-chased records are separated from database hits in Figure 1. Some eligible papers never use “laparoscop*” in the title or abstract. Others sit in journals the registered databases do not index. Both are among the 64 reports in Table 2.

Selection of Sources

Screening had two stages (title/abstract, then full text). Screeners were not blinded to authors, journal, or year. Records without a full text were retained on the abstract unless later excluded; fields that needed the body were coded “full text not obtained.”

The second author screened all 329 Ichushi-Web records independently. For 40 of the records, the first author had not yet recorded a decision when that workbook was returned, so those 40 were not screened blind and are left out of the agreement calculation. The first author resolved the remaining disagreements against the written PCC criteria; the second author had excluded case reports and conference records on design grounds, and that exclusion was not adopted. The third author was named to arbitrate. Cohen’s κ is therefore reported only for the 289 records that both authors screened independently, and it does not apply to PubMed or to citation chasing.

The first author screened the PubMed and citation-chased records alone. Eligible originals are the main analysis; eligible conference records are a supplementary layer. Two Ichushi originals tagged at dual screen were later removed because diagnostic laparoscopy inspected GTD without resection or biopsy. Four records first flagged as citation-chased were recoded to PubMed as hits of the registered string.

Data Charting

The first author was the sole extractor and completed a pre-specified form; duplicate extraction was not performed. Rules are written to named fields, tallies are scripted, and a dated log records each rule. Where abstract and body disagreed, the body was used.

Reports with more than one GTD-targeted operation were split into one row per operation. Report-level fields (including decision, publication type, source, journal domain, spillage-word mention, retrieval status) were counted only on the representative row. Units of analysis are reports, operations (preoperative-diagnosis axis), and cases (patient characteristics).

Absence was not collapsed: not reported (silent; stays in the denominator); not applicable (the operation cannot raise the question; out of the denominator); full text not obtained (out of the denominator). No values were imputed. After the full text was in hand, remaining silences were “not reported.”

Video articles have no body text. Their technical items were coded “not reported.” The spillage-word field was also coded “not reported” for them, as an exception to the rule that the spillage-word search needs a full text. Two Journal of Reproductive Medicine reports could not be obtained; the journal ceased publication in 2021, and its archive is no longer reachable. One further report is incomplete due to missing pages. Full-text pursuit was closed for nine further main analysis reports that remained abstract-only; they stay in the main analysis because Concept was met on the abstract. These groups are identified in the Results section.

Technical items include pneumoperitoneum pressure, uterine manipulator, proximal uterine artery control, tubal occlusion, extraction route, retrieval bag, morcellation, and peritoneal washing cytology. A report counts as describing a containment technique when at least one of the following coded values appears. We did not infer the surgeon’s intent. The values are manipulator explicitly not used; proximal uterine artery ligation, preoperative embolization, or other ligation; vaginal extraction; retrieval bag used; morcellation explicitly not performed; and washing cytology performed. Usual-site uterine artery division at hysterectomy does not score. Explicit mention of spillage risk is independent of containment and requires the words spillage, seeding, dissemination, or port-site. Each extraction cell begins with a coded value and may be followed by free text in parentheses. Only the coded value at the start of the cell is counted; the text in parentheses is kept as narrative source material and never enters a numerator or a denominator.

Journal domain is from the journal title. The preoperative-diagnosis axis is 1a (GTD diagnosed before the index operation), 1b-i (not diagnosed; no oncological sampling), 1b-ii (not diagnosed; oncological sampling), or 1b unclassifiable. Denominators differ by item because not every operation can raise every question. The uterine manipulator, proximal uterine artery control, the extraction route, and morcellation are counted only on operations in which the uterus was still present. The retrieval bag and peritoneal washing cytology are counted on those operations and on operations for GTD at an ectopic site as well. Pneumoperitoneum pressure and tubal occlusion can be described in any operation, so their denominators follow the coded value alone. Metastatic or recurrent deposits of uterine-origin GTD are not ectopic-site GTD.

Synthesis

Synthesis is descriptive: counts, reporting rates, and cross-tabulations. A reporting rate is descriptions divided by reports in which the item could be described; “not applicable” and “full text not obtained” are out of the denominator, so denominators differ by item. No inferential statistics, domain comparisons, or pooled safety or effectiveness estimates were produced. Individual sources were not critically appraised, consistent with JBI guidance for a scoping review of what is reported [12]. Our conclusions are limited to what this literature does and does not report, and to the citation relationships between reports. We draw no conclusion that any technique prevents spillage, and reports that contradict the descriptive pattern are presented alongside it.

Deviations After Registration

The following changes were made after the OSF snapshot was taken: (1) Five further PubMed reports that met Concept were extracted. (2) Abstracts in which Concept was uncertain and whose full text was not obtained were removed from the main analysis. (3) Two Ichushi-Web reports included at dual screening, and one PubMed report in which laparoscopy only inspected the disease, were removed because they did not meet Concept. (4) One report was excluded as non-gestational. (5) Full-text pursuit was closed for nine main analysis reports. (6) Four records were recoded from citation chasing to the database that had retrieved them. (7) One report found only through the reference list of an article was added to the main analysis. (8) Cohen’s κ was computed on the independently dual-screened Ichushi-Web subset. An earlier value of about 0.20, calculated on 118 records, is not used. (9) Citation searching was extended electronically after the main analysis was closed, as described under Information Sources and Search. It found no further eligible reports.

Results

Selection of Sources

PubMed (August 18, 2026) returned 449 records at registration; a dated re-run of the same string returned 448, one registered hit having been lost to an indexing change. Ichushi-Web (August 20, 2026) returned 329 records, three of which had also been retrieved by PubMed and were counted once on the PubMed side. Backward citation chasing identified two further eligible reports that neither string retrieved.

The 448 PubMed records were screened by the first author alone, and 403 were excluded: six were search false positives, the string retrieved records in which ETT denotes endotracheal tube or GTN denotes nitroglycerin, and 397 failed the PCC or protocol criteria (§5.1-§5.3, Concept, study design). Almost all of the 397 were decided on the title and abstract; two were settled only after the full text had been read. In total, 45 PubMed reports were included. On the Ichushi-Web side, 326 records were screened after the three cross-database duplicates were removed, 285 were excluded, and 17 originals unique to Ichushi-Web and 24 conference records remained.

Finally, 64 original reports [15-78] entered the main analysis: 45 from PubMed, 17 from Ichushi-Web, and two citation-chased reports. They contribute 67 operations, because three reports were split. The 24 conference records form a supplementary layer.

In the reference list, [15-78] are these 64 included reports. References [1-14] are guidelines, methodological sources, and our registered protocol, and [79,80] are the two adjacent reports discussed later but not included.

Two sets of counts in this review are not interchangeable. Figure 1 counts identification streams, whereas Table 1 counts the source field of the extraction sheet: two of the three cross-database reports (PMID 33078868 and 40698231) are coded Ichushi-Web there, and the third (Jiang et al., 2018 [48]) is coded PubMed, so Figure 1’s 45 and 17 become Table 1’s 43 and 19. A third figure belongs to neither: the Ichushi-Web dual-screen protocol sheet tagged 18 originals, two of which were excluded at full text, and those 18 are interchangeable with neither the extraction-sheet 19 nor the 17 Ichushi-only originals in Figure 1. Separately, the screened set is not the registered yield, and the adjacent reports collected for the Discussion are not screening exclusions; both points are set out in the Methodology section and in the Figure 1 legend.

**Table 1:** Characteristics of the 64 main analysis reports and the 67 operations they describe. Each row is one count. Rows marked (operations) sum to 67; all other rows in a block sum to 64. Retrieval status describes how much of each report was available for charting.

Characteristic	n
Publication year 1990–1999	1
Publication year 2000–2009	4
Publication year 2010–2019	34
Publication year 2020–2026	25
Source: PubMed	43
Source: Ichushi-Web	19
Source: citation chasing only	2
Journal domain: general obstetrics and gynecology	33
Journal domain: non-gynecological	25
Journal domain: gynecologic oncology	6
Design: case report	54
Design: series	6
Design: video	3
Design: retrospective comparison	1
Retrieval: full text in hand	49
Retrieval: abstract only, pursuit closed	9
Retrieval: video	3
Retrieval: journal ceased	2
Retrieval: incomplete pagination	1
Approach: conventional laparoscopy (operations)	64
Approach: robot-assisted (operations)	3
Operative target: uterus (operations)	44
Operative target: ectopic or extrauterine (operations)	23

The Ichushi-Web records were screened independently by a second reviewer, on all 329 as retrieved rather than on the de-duplicated 326. Agreement on the subset screened independently by both (n = 289) was 87.5% (both include five; first author includes and second screener excludes 15; first author excludes and second screener includes 21; both exclude 248). Cohen’s κ was 0.151, and the prevalence-adjusted, bias-adjusted kappa was 0.75. The gap between the two is the kappa paradox: 86% of the records were excluded by both screeners, so expected agreement is high, and κ is driven down, although the screeners differed on only 36 of the 289 records. Those differences were systematic rather than random, turning on whether conference records and case reports are design-ineligible, and were resolved in favor of the protocol.

A complete full text was in hand for 49 of the 64 reports. Full-text pursuit was closed for nine that remain abstract-only (including Ji et al. (2010) [21]); two reports in the Journal of Reproductive Medicine could not be obtained, the journal having ceased publication; one is incomplete through missing pages [36]; and three are video articles, coded under the video rule.

Characteristics of Sources

The 64 reports span 1995 to 2026, 59 of them published from 2010 onward. Most appeared in general obstetrics and gynecology journals or in non-gynecological journals rather than in gynecologic oncology, and most are case reports, six of which also carry a narrative or literature review (Table 1). Each of the 64 reports is cited and summarized in Table 2.

**Table 2:** Included reports of laparoscopic or robot-assisted surgery for GTD. Each row is one of the 64 main analysis reports. Sample size is the number of eligible patients in that report and is not a pooled total. For the retrospective comparison, sample size is the minimally invasive arm (n = 22); the 17 open hysterectomies are outside the review question. Key findings name the operative procedure and the charted technical items that the report described (uterine manipulator, uterine-artery handling, tubal occlusion, extraction route, retrieval bag, morcellation, peritoneal washing cytology). Silence, not applicable, and full text not obtained are not listed as findings. These cells are not outcome estimates. Axis 1a: GTD diagnosed before the index operation; 1b-i: not diagnosed beforehand and no oncological sampling; 1b-ii: not diagnosed beforehand, with oncological sampling. Japanese-language reports that PubMed does not index are cited by Ichushi accession in the reference list. ETT: epithelioid trophoblastic tumor; GTD: gestational trophoblastic disease; GTN: gestational trophoblastic neoplasia; PSTT: placental site trophoblastic tumor

Reference	Study design	Sample size	Study population	Key findings
Lang et al. (1995) [15]	Case report	1	GTN; axis 1a; uterine target; conventional laparoscopy	Full text not obtained. Laparoscopic hysterectomy. Described: uterine arteries divided before uterine handling; morcellation omitted
Nayama et al. (2007) [16]	Case report	1	Choriocarcinoma; axis 1b-i; extrauterine target; conventional laparoscopy	Laparoscopic right salpingectomy. No charted technical item was described
Rotas et al. (2007) [17]	Case report	1	Choriocarcinoma; axis 1b-i; uterine target; conventional laparoscopy	Diagnostic laparoscopy then left cornual wedge resection in the same sitting. No charted technical item was described
Noh et al. (2008) [18]	Case report	1	ETT; axis 1b-ii; uterine target; conventional laparoscopy	Laparoscopic hysterectomy with bilateral salpingo-oophorectomy, peritoneal biopsy, and later second-look laparoscopy. Described: peritoneal washing cytology performed
Corusic et al. (2009) [19]	Case report	1	Invasive mole; axis 1a; uterine target; conventional laparoscopy	Full text not obtained. Laparoscopic tumor resection with bipolar coagulation of feeding arterial branches and uterine-wall repair. Described: bipolar coagulation of feeding arterial branches
Hwang et al. (2010) [20]	Case report	1	Partial mole; axis 1b (unclassifiable); uterine target; conventional laparoscopy	Full text not obtained. Laparoscopic cornuostomy. No charted technical item was described
Ji et al. (2010) [21]	Case report	1	Complete mole; axis 1b-i; extrauterine target; conventional laparoscopy	Full text not obtained. Laparoscopic left salpingectomy. No charted technical item was described
Namaky et al. (2010) [22]	Case report	1	PSTT; axis 1a; uterine target; robot-assisted	Robot-assisted hysterectomy with pelvic washings. Described: uterine manipulator not used; peritoneal washing cytology performed. Spillage-word present
Takahama et al. (2012) [23]	Case report	1	Choriocarcinoma; axis 1a; uterine target; conventional laparoscopy	Semi-urgent laparoscopic hysterectomy after two preoperative uterine-artery embolization procedures. Described: preoperative uterine-artery embolization
Shen et al. (2012) [24]	Case series	1	PSTT; axis 1a; uterine target; conventional laparoscopy	Laparoscopic uterine tumor resection after hysteroscopic exploration (one of six PSTT cases). No charted technical item was described
Moreno-Collado et al. (2012) [25]	Case report	1	Choriocarcinoma; axis 1a; uterine target; conventional laparoscopy	Laparoscopic hysterectomy. Described: uterine manipulator not used; uterine arteries taken at origin; tubal occlusion performed. Spillage word present
Bousfiha et al. (2012) [26]	Case report	1	Partial mole; axis 1b-i; extrauterine target; conventional laparoscopy	Diagnostic laparoscopy then laparoscopic left salpingectomy. No charted technical item was described
Hassadia et al. (2012) [27]	Case series	12	Choriocarcinoma; axis 1b (unclassifiable); extrauterine target; conventional laparoscopy	Full text unobtainable (journal ceased). Ectopic-GTD series; route mix among the 12 confirmed cases is not stated in the abstract. No charted technical item was described
Scott et al. (2012) [28]	Case report	1	ETT; axis 1b-i; uterine target; conventional laparoscopy	Laparoscopically assisted vaginal hysterectomy with adnexal surgery. Described: vaginal extraction; peritoneal washing cytology performed
Koike et al. (2013) [29]	Case report	1	Invasive mole; axis 1a; uterine target; conventional laparoscopy	Emergency laparoscopy for hemoperitoneum, with the uterus handled. No charted technical item was described
Huang et al. (2013) [30]	Case report	1	Complete mole; axis 1b-i; extrauterine target; conventional laparoscopy	Two operations: laparoscopic right salpingectomy with endometrial curettage, then later local pelvic resection. No charted technical item was described. Spillage word present
Funayama et al. (2014) [31]	Case report	1	Molar tissue; axis 1b-i; extrauterine target; conventional laparoscopy	Laparoscopic right salpingotomy with tubal conservation. No charted technical item was described
Kitagawa et al. (2014) [32]	Case report	1	Choriocarcinoma; axis 1a; extrauterine target; conventional laparoscopy	Laparoscopic right adnexectomy. No charted technical item was described
Fang et al. (2014) [33]	Case report	1	ETT; axis 1b-i; uterine target; conventional laparoscopy	Diagnostic laparoscopy followed by laparotomy for complete mass resection. No charted technical item was described
Meddeb et al. (2014) [34]	Case report	1	Choriocarcinoma; axis 1b-i; uterine target; conventional laparoscopy	Laparoscopic local resection, then emergency laparotomy hysterectomy one month later. No charted technical item was described
Doi et al. (2015) [35]	Case report	1	Choriocarcinoma; axis 1a; extrauterine target; conventional laparoscopy	Laparoscopic right salpingectomy. No charted technical item was described
Riley et al. (2015) [36]	Case report	1	Complete mole; axis 1a; uterine target; robot-assisted	Incomplete pagination. Robot-assisted hysterectomy. No charted technical item was described
Yao et al. (2015) [37]	Case report	1	Invasive mole; axis 1a; uterine target; conventional laparoscopy	Laparoscopic hysterectomy with concurrent open left nephrectomy. No charted technical item was described
Li et al. (2015) [38]	Case series	17	Invasive mole; axis 1a; uterine target; conventional laparoscopy	Full text not obtained. Laparoscopy for suspected uterine GTN; procedure mix not stated in the abstract. No charted technical item was described
Kawai et al. (2016) [39]	Case report	1	ETT; axis 1a; uterine target; conventional laparoscopy	Total laparoscopic hysterectomy after inspection for peritoneal deposits. No charted technical item was described
Ikoma et al. (2017) [40]	Case report	1	Invasive mole; axis 1a; uterine target; conventional laparoscopy	Total laparoscopic hysterectomy. No charted technical item was described
Natsume et al. (2017) [41]	Case report	1	Invasive mole; axis 1a; uterine target; conventional laparoscopy	Laparoscopic hysterectomy with salpingectomy. No charted technical item was described
Grin et al. (2017) [42]	Video article	1	Invasive mole; axis 1b (unclassifiable); uterine target; conventional laparoscopy	Video article. Laparoscopic local resection. No charted technical item was described
Chen et al. (2017) [43]	Case report	1	Complete mole; axis 1a; uterine target; conventional laparoscopy	Laparoscopic cornuostomy with vasopressin injection. Described: vasopressin injection
Liu et al. (2017) [44]	Case series	5	Choriocarcinoma; axis 1b-i; uterine target; conventional laparoscopy	Laparoscopic uterine wedge resection with frozen section (five presumed cornual pregnancies). No charted technical item was described
Nie et al. (2017) [45]	Case report	1	PSTT; axis 1a; uterine target; conventional laparoscopy	Full text not obtained. Laparoscopic hysterectomy with ovarian conservation. No charted technical item was described
Lu et al. (2017) [46]	Case series	4	Choriocarcinoma; axis 1b-i; extrauterine target; conventional laparoscopy	Full text unobtainable (journal ceased). Laparoscopic surgery in all four tubal GTD cases; individual procedures not stated in the abstract. No charted technical item was described
Matsuki et al. (2017) [47]	Case series	1	Choriocarcinoma; axis 1b-i; extrauterine target; conventional laparoscopy	Full text not obtained. Diagnostic laparoscopy then partial omentectomy (second of two cases; only this case was GTD). No charted technical item was described
Jiang et al. (2018) [48]	Case report	1	ETT; axis 1b-i; extrauterine target; conventional laparoscopy	Laparoscopic local resection. Described: retrieval bag used
Aloysius et al. (2018) [49]	Case report	1	Choriocarcinoma; axis 1b-i; extrauterine target; conventional laparoscopy	Two operations: emergency diagnostic laparoscopy with evacuation and bilateral tubal ligation at the patient’s request, then emergency laparoscopic splenectomy. Described: tubal ligation at the patient’s request (not coded as an oncological maneuver)
Mochii et al. (2019) [50]	Case report	1	Choriocarcinoma; axis 1b-i; uterine target; conventional laparoscopy	Emergency laparoscopic left salpingectomy then hysterectomy in the same sitting. No charted technical item was described
Takahashi et al. (2019) [51]	Case report	1	Exaggerated placental site; axis 1a; uterine target; conventional laparoscopy	Laparoscopically assisted vaginal hysterectomy. No charted technical item was described
Ferrier et al. (2019) [52]	Video article	1	ETT; axis 1a; extrauterine target; conventional laparoscopy	Video article. Laparoscopic pelvic cytoreduction. No charted technical item was described
Lee et al. (2019) [53]	Case report	1	Invasive mole; axis 1a; uterine target; conventional laparoscopy	Laparoscopic local resection. Described: port extraction; retrieval bag used. Spillage word present
Yamazoe et al. (2020) [54]	Case report	1	Invasive mole; axis 1b-i; uterine target; conventional laparoscopy	Total laparoscopic simple hysterectomy. No charted technical item was described
Katayama et al. (2019) [55]	Case report	1	Choriocarcinoma; axis 1b-i; extrauterine target; conventional laparoscopy	Laparoscopic partial omentectomy. No charted technical item was described
Rey et al. (2020) [56]	Case report	1	PSTT; axis 1a; uterine target; conventional laparoscopy	Laparoscopic hysterectomy with bilateral salpingectomy. No charted technical item was described
Suzuki et al. (2021) [57]	Case report	1	Molar tissue; axis 1b-i; extrauterine target; conventional laparoscopy	Laparoscopically assisted resection of a fundal serosal lesion. No charted technical item was described
Patel et al. (2021) [58]	Case report	1	Complete mole; axis 1a; uterine target; conventional laparoscopy	Laparoscopic hysterectomy. Described: uterine manipulator not used; uterine arteries divided at the usual site; tubal occlusion performed; vaginal extraction; morcellation omitted
Sugrue et al. (2021) [59]	Retrospective comparison	22	Mixed GTD histology; axis 1a; uterine target; conventional laparoscopy	Minimally invasive hysterectomy (open arm not eligible). Described: uterine manipulator used. Spillage word present
Bakır et al. (2021) [60]	Case report	1	Choriocarcinoma; axis 1a; uterine target; conventional laparoscopy	Laparoscopic hysterectomy. Described: uterine arteries taken at origin; tubal occlusion performed; peritoneal washing cytology performed
Hayashi et al. (2022) [61]	Case report	1	Choriocarcinoma; axis 1a; uterine target; conventional laparoscopy	Laparoscopic hysterectomy with bilateral salpingectomy. No charted technical item was described
Du et al. (2022) [62]	Case report	1	Choriocarcinoma; axis 1a; extrauterine target; conventional laparoscopy	Emergency laparoscopy then nephron-sparing partial nephrectomy. No charted technical item was described
Kriplani et al. (2022) [63]	Video article	1	Partial mole; axis 1b-i; uterine target; conventional laparoscopy	Video article. Laparoscopic local resection of a caesarean scar lesion. No charted technical item was described
D’Asta et al. (2022) [64]	Case report	1	Invasive mole; axis 1b-i; extrauterine target; conventional laparoscopy	Laparoscopic right salpingectomy. No charted technical item was described
Karafezieva et al. (2023) [65]	Case report	1	Choriocarcinoma; axis 1a; uterine target; robot-assisted	Robot-assisted type II radical hysterectomy with bilateral salpingo-oophorectomy and selective pelvic lymphadenectomy. Described: uterine manipulator not used; uterine arteries taken at origin
Oishi et al. (2024) [66]	Case report	1	PSTT; axis 1a; uterine target; conventional laparoscopy	Two operations: laparoscopic left cornual wedge resection, then total laparoscopic hysterectomy. No charted technical item was described
Furui et al. (2024) [67]	Case report	1	ETT; axis 1a; uterine target; conventional laparoscopy	Laparoscopic hysterectomy with bilateral salpingectomy. Described: vaginal extraction
Huang et al. (2024) [68]	Case report	1	ETT; axis 1a; uterine target; conventional laparoscopy	Laparoscopic hysterectomy with bilateral salpingectomy. Described: vaginal extraction; morcellation omitted. Spillage-word present
Ishii et al. (2025) [69]	Case report	1	PSTT; axis 1a; uterine target; conventional laparoscopy	Laparoscopic hysterectomy with bilateral salpingectomy. Described: peritoneal washing cytology performed
Fathaddin (2025) [70]	Case report	1	Complete mole; axis 1b-i; extrauterine target; conventional laparoscopy	Laparoscopic right salpingectomy and ovarian cystectomy. No charted technical item was described
Maekawa et al. (2025) [71]	Case report	1	Choriocarcinoma; axis 1b-i; extrauterine target; conventional laparoscopy	Laparoscopic resection of an ileocecal peritoneal deposit. No charted technical item was described
Kamada et al. (2025) [72]	Case report	1	ETT; axis 1a; uterine target; conventional laparoscopy	Full text not obtained. Laparoscopic hysterectomy with bilateral salpingo-oophorectomy. Described: peritoneal washing cytology performed
Wang et al. (2026) [73]	Case report	1	Molar tissue; axis 1a; extrauterine target; conventional laparoscopy	Full text not obtained. Laparoscopy; procedure mix not stated in the abstract. No charted technical item was described
Xiang et al. (2026) [74]	Case report	1	ETT; axis 1b-i; uterine target; conventional laparoscopy	Laparoscopic simple hysterectomy with bilateral salpingo-oophorectomy after preoperative uterine artery embolization. Described: preoperative uterine artery embolization
Ting (2026) [75]	Case report	1	ETT; axis 1a; uterine target; conventional laparoscopy	Laparoscopic hysterectomy with bilateral salpingectomy. No charted technical item was described
Uysal (2026) [76]	Case report	1	Complete mole; axis 1a; uterine target; conventional laparoscopy	Laparoscopic hysterectomy with bilateral salpingo-oophorectomy. No charted technical item was described
Xu et al. (2026) [77]	Case report	1	Invasive mole; axis 1b-i; uterine target; conventional laparoscopy	Laparoscopic left cornual wedge resection with uterine reconstruction and bilateral uterine artery ligation. Described: bilateral uterine artery ligation
Ogawa et al. (2026) [78]	Case report	1	Partial mole; axis 1b-i; extrauterine target; conventional laparoscopy	Laparoscopic right salpingectomy. No charted technical item was described

Six reports were case series. In two of these, only one case was eligible: a two-case report of omental pregnancy in which only the second case was GTD (Ichushi 2017351213; Table 2), and Shen et al. (2012) in which only one of six PSTT cases underwent laparoscopic tumor resection [24]. Each therefore contributes a single patient, and both are counted as single-patient reports in the next subsection.

Three of the 67 operations were robot-assisted: hysterectomy for chemorefractory myoinvasive complete mole [36], hysterectomy for intramural PSTT [22], and radical hysterectomy for cervical choriocarcinoma [65]. The other 64 were conventional laparoscopy. The uterus was the operative target in 44 operations (43 reports) and an ectopic implantation site or extrauterine deposit in 23 (21 reports). Country was not a coded field; reports were read in English, Japanese, or Chinese as published. These characteristics are summarized in Table 1.

Pathology and Size of the Underlying Case Population

A total of 59 reports described a single eligible patient. Their leading coded diagnoses were gestational choriocarcinoma (n = 16), invasive mole (n = 10), ETT (n = 11), complete mole or molar tissue (n = 9), PSTT (n = 6), partial mole (n = 4), exaggerated placental site (n = 1), clinical GTN/persistent trophoblastic disease (n = 1), and parametrial mole (n = 1). FIGO stage was not reported in 31 of these 59 reports.

One of the 59 was a tumor-like lesion (exaggerated placental site; Ichushi 2020046307, Table 2). It was retained because it could not be distinguished from PSTT before the specimen was examined; the planned sensitivity analysis that drops it is reported under Sensitivity Analyses.

The remaining five reports, four case series and the comparison, describe groups of patients whose individual operations are not separately described. Each is counted as one report in the totals above, and their patient counts are not added into a single-patient total (Table 3).

**Table 3:** Four case series and one retrospective comparison among the 64 reports, with patient counts not pooled. These five reports are also rows in Table 2. Each is counted once in the main analysis; the patient numbers shown are not added into a single-patient total. For Sugrue et al. [59], Table 3 records the paper’s 39 hysterectomies (22 minimally invasive, 17 open); Table 2 sample size is the eligible minimally invasive arm only. GTD: gestational trophoblastic disease; GTN: gestational trophoblastic neoplasia; PSTT: placental site trophoblastic tumor

Report	What can be counted
Lu et al., 2017 [46]	4 tubal GTD cases from a registry of 619
Hassadia et al., 2012 [27]	12 confirmed ectopic GTD among 42 suspected
Li et al., 2015 [38]	17 confirmed GTN among 62 suspected (choriocarcinoma 8, invasive mole 5, PSTT 4)
Liu et al., 2017 [44]	5 cornual choriocarcinomas, 2010–2014
Sugrue et al., 2021 [59]	39 hysterectomies for GTD, of which 22 were minimally invasive and 17 open

Preoperative-Diagnosis Axis (Operations)

The axis was coded on all 67 operations (Table 4). Most axis-1a operations were on the uterus (31 of 38), and most axis-1b-i operations were not (15 of 25), being directed at tubal, cornual, omental, or other extrauterine targets treated as ectopic pregnancy or another benign condition. The single 1b-ii operation was a paracervical/parametrial ETT in which peritoneal biopsy was the sampling step [18]; the three unclassifiable operations are the 2012 ectopic-GTD series, a video article, and the abstract-only Hwang et al. (2010) [27,42,20]. One report supplies an operation on each of two axes, an emergency laparoscopy coded 1b-i and a later splenectomy coded 1a, and both are counted [49].

**Table 4:** Preoperative-diagnosis axis (67 operations). Counts are operations, not reports; one report contributes an operation to each of two axes. Axis 1a means GTD was diagnosed before the index operation and 1b that it was not, subdivided by whether oncological tissue sampling was performed. GTD: gestational trophoblastic disease

Axis	Definition	Operations
1a	GTD diagnosed before the index operation	38
1b-i	GTD not diagnosed; no oncological tissue sampling	25
1b-ii	GTD not diagnosed; oncological sampling performed	1
1b Unclassifiable	GTD not diagnosed; sampling status not decidable from the Report	3

Technical Item Reporting

The expectation recorded before extraction, that these items are rarely described, is confirmed on the main analysis rather than on the nine-report pilot (Table 5).

**Table 5:** Reporting of technical items (64 reports; 67 operations as sensitivity). Each cell shows the number of records in which the item was described, divided by the number in which it could have been described, with the percentage in parentheses. “Not reported” remains in the denominator because silence is the finding; “not applicable” and “full text not obtained” are excluded, so denominators differ by item. Video articles keep their technical items as “not reported.” These are rates of description, not rates of practice.

Item	Reports, described/denominator (%)	Operations, described/denominator (%)
Uterine manipulator	5/36 (13.9)	5/37 (13.5)
Proximal uterine artery control (any coded action)	10/37 (27.0)	10/38 (26.3)
Tubal occlusion	4/30 (13.3)	4/31 (12.9)
Extraction route	5/35 (14.3)	5/36 (13.9)
Retrieval bag	2/50 (4.0)	2/53 (3.8)
Morcellation (performed or explicitly avoided)	3/36 (8.3)	3/37 (8.1)
Peritoneal washing cytology	6/53 (11.3)	6/56 (10.7)
Pneumoperitoneum pressure	0/52 (0)	0/55 (0)

Denominators differ by item and follow the Methodology: “not reported” stays in, “not applicable” and “full text not obtained” are out, and video articles keep technical items as “not reported.” Manipulator, artery, extraction route, and morcellation are counted on operations with a retained uterus; bag, washing, pneumoperitoneum, and tubal occlusion follow the cell. A split report adds an operation to a denominator only where the additional operation can raise that question.

Every numerator in Table 5 is small enough to name. The manipulator was mentioned in five reports, and in four of these, only to say that none was used [25,22,65,58]; the fifth used one [59]. A retrieval bag was described in two [48,53], and pneumoperitoneum pressure in none. Of the four reports describing tubal occlusion, one was a bilateral tubal ligation performed at the patient’s request during a non-uterine emergency laparoscopy [49]; it is not an oncological maneuver but remains in the numerator because the action was described.

The artery item has the largest and most heterogeneous numerator. The 10 described actions were origin ligation (n = 3), preoperative uterine artery embolization (n = 2), and one each of ligation at the usual hysterectomy site, bilateral uterine artery ligation, division before uterine manipulation, bipolar coagulation of feeding branches, and vasopressin injection; only some of these are containment triggers.

Reporting of the manipulator was uncommon in every journal domain: one of 16 evaluable general obstetrics and gynecology reports, three of 15 non-gynecological, and one of five gynecologic oncology. Domains were not compared statistically, and the smallest denominator cannot support an interpretation.

Reporting was also examined by era. Splitting the 64 reports at 2020, the year of the first practical guideline to mention a laparoscopic approach [7], gives 39 reports through 2019 and 25 afterwards. Most technical items, the containment token, and the port-site field were described more often in the later group, but the retrieval bag fell from two of 28 evaluable reports to none of 22, one of the four spillage words from four of 31 to two of 23, and pneumoperitoneum pressure was absent in both eras. The eras were not compared statistically.

Containment and Explicit Mention of Spillage Risk

Two variables were coded independently. Containment, derived from mechanical tokens without inferring intent, was evaluable in 57 reports and present in 17; because it is built from the same coded fields that Table 5 counts, it summarizes those fields rather than adding an independent measurement. A containment token was present in 12 of the 31 evaluable reports whose operations were all 1a, in four of the 21 whose operations were all 1b-i, and in the single 1b-ii report [18]; the remaining four evaluable reports had none: two describe operations on both axes [30,49], and two could not be classified. These counts were not compared statistically. Explicit mention, defined as the words spillage, seeding, dissemination, or port-site, was evaluable in 54 reports and present in six: a pelviscopic resection that used a bag to prevent seeding [53]; laparoscopic resection of recurrent intraperitoneal choriocarcinoma [30]; a uterine ETT managed with vaginal extraction and no morcellation [68]; robotic hysterectomy for PSTT [22]; Sugrue et al. (2021) [59]; and a laparoscopic hysterectomy for choriocarcinoma performed without uterine manipulation [25]. Mentions of embolization or of tumor rupture were not counted. The two variables are crossed in Table 6.

**Table 6:** Described containment by explicit spillage word mention (54 reports with both axes evaluable). Counts are reports. Containment is a derived binary scored from coded technical values without inferring the surgeon's intent. Explicit mention is present only when the words spillage, seeding, dissemination, or port-site appear in the report. “Mention not reported (video)” identifies video articles, which have no body text, so the word search could not be run on them. Three containment-evaluable reports without a full text are excluded from this table.

Containment	Mention present	Mention absent	Mention not reported (video)
Containment present	4	12	0
Containment absent	2	33	3

Table 6 crosses the two variables on the 54 reports evaluable for both, among them 16 of the 17 in which containment was present. They rarely coincide: four reports describe a containment maneuver and use one of the words, 12 describe a maneuver without using any of them, two use a word without any coded maneuver (Huang et al. (2013) [30] and Sugrue et al. (2021) [59]), and the remaining 36 do neither.

Table 6 excludes three containment-evaluable reports that lack a full text: an abstract-only ETT report whose mention field remains “full text not obtained” (Table 2) and the two Journal of Reproductive Medicine series [46,27]. Containment can be derived from technical items that an abstract supplies, whereas the spillage word search requires the full text.

Re-deriving “containment present” from the trigger list in the Methodology section reproduced the stored code on all 57 evaluable reports (17 present, 40 absent). Bilateral uterine artery ligation was counted as other ligation [77]; vasopressin [43], bipolar coagulation of feeding branches after resection [19], and uterine-artery division at the usual hysterectomy site were not.

Outcomes That Were Charted but Not Pooled

Conversion to laparotomy, blood loss, human chorionic gonadotropin (hCG) resolution, recurrence, death, and port-site events were extracted when the text stated them. None of these are reported here as a rate. The exception is the port-site field, where the extent of silence is itself a finding of this review: port-site events were charted as an outcome field; that field was evaluable in 49 reports and was “not reported” in 43 of them. No conclusion is drawn that any technique is safe or prevents dissemination.

Supplementary Conference Records

A total of 24 eligible Ichushi-Web conference records were set aside as a supplementary layer (Appendix B). They are not included in Table 4 or Table 5.

Sensitivity Analyses

Three sensitivity analyses were registered, each against a coding decision a reader might contest; two were carried out.

The first tests the inclusion of a tumor-like lesion in a review of GTD. Dropping the exaggerated placental site report leaves 63 reports; no Table 5 numerator changes, each denominator in which it was evaluable falls by one, and the manipulator finding becomes five of 35 (14.3%).

The second tests the unit of analysis, which is ambiguous because three reports describe more than one operation. Report-level and operation-level rates differ by less than one percentage point (Table 5). Rank order is the same at both levels.

The third would have tested the denominator rules, through alternative denominators for retrieval bag and peritoneal washing, and was not carried out (see Limitations). This matters more than either analysis that was run: both move the reported rates by less than one percentage point, whereas the manipulator’s five of 36 evaluable reports of operations on a retained uterus (13.9%) would be 7.5% over all 67 operations.

Discussion

What This Review Can Say

The 64 original reports covering 67 operations answer the review’s question at the level of reporting, and only at that level. Most are single-patient case reports; four case series and one comparison cannot be collapsed into a common patient denominator; and the technical items needed to judge containment are undescribed in the large majority of evaluable reports. A percentage built from conversion, hemorrhage, hCG resolution, recurrence, or death would describe the reporting habits of a small and heterogeneous group of authors rather than a surgical risk. No estimate of whether the laparoscopic route is safe or effective in GTD is offered.

Who Is Being Operated On

Two different clinical decisions sit behind these 67 operations. A planned hysterectomy for PSTT or ETT and an emergency salpingectomy for a presumed ectopic pregnancy that is later read as molar tissue or choriocarcinoma share a specimen and a route, but not a preoperative oncological plan. Table 4 separates them: GTD was anticipated before 38 operations and not before 29. The distinction matters for what this review measures, because containment can be planned in advance only when GTD is anticipated. Aloysius and Shelat show the extreme case. The first laparoscopy was undertaken for a presumed ruptured ectopic pregnancy at an hCG of 508,017 mIU/mL, and GTD was established only when imaging later demonstrated a ruptured splenic metastasis [49].

The disease reaching laparoscopy is also not the typical GTN of the guidelines. Overall, 17 of the 59 single-patient reports are PSTT or ETT, and both are entities in which surgery is primary rather than salvage therapy [2,7]. Stage cannot be used to stratify this literature: it was not reported in 31 of the 59.

Robot-assisted operations, although named in the review’s question, appear in only three reports [36,22,65]; they were counted with laparoscopy, as registered, and are too few to form a separate technique class.

What Is Not Written

These are rates of description, not rates of practice. “Not reported” stayed in every denominator, so an author who used a uterine manipulator and did not say so is counted with one who did not use one. The same caution applies to the one item that is not sparse: the 10 of 37 for proximal uterine artery control counts every described vessel action, including divisions at the usual hysterectomy site, which carry no containment intent. At the other extreme, no report gave the pneumoperitoneum pressure: none of 52, in any report since 1995.

Containment tokens were more common in reports where every operation was 1a (12 of 31) than where every operation was 1b-i (4 of 21). The denominators are small, and the counts were not compared statistically, so this is a description and not an effect; but the direction is what one would expect of a maneuver that has to be planned in advance. The review makes no claim that one specialty reports better than another. Reporting has not simply improved with time: the era split shows most items described more often after 2020, but the retrieval bag and the spillage words moving the other way.

What these maneuvers lack is a stated reason. Of the 17 reports with a containment token, 16 could also be assessed for wording, and 12 of those never used the words spillage, seeding, dissemination, or port-site. Two of the six reports that did use one of those words recorded no containment maneuver at all, and one of the two is the single comparative series, whose authors wrote that the importance of these maneuvers is unproven and unclear [59].

The same gap between action and reason runs through the artery field. One report describes bilateral uterine artery ligation, and gives the reason as reducing postoperative bleeding [77]. An earlier report describes the same kind of proximal ligation, and gives the reason as preventing tumor rupture and bleeding [60]. The later report does not cite the earlier one. A closely similar proximal ligation is therefore described twice, for two different reasons, by authors who share no technical vocabulary. That is a citation relationship, not evidence that ligation contains trophoblast.

Reports That Cannot Bear on Dissemination

Two sets of reports are often used to argue that laparoscopy disseminates GTD. Neither can support that argument. The first is the literature on histologically normal villus found on the peritoneum after surgery for ectopic pregnancy [79,80]. It was collected because it supplies the mechanism people have in mind when they worry about trophoblast seeding a port site or a peritoneal surface. But it is not GTD: residual villus after salpingotomy is a different disease, a different denominator, and a different operation. It is cited here to mark the edge of the review, not to report an outcome.

The second is two included reports in which hCG rose quickly after laparoscopic resection of a mass that proved to be choriocarcinoma, one interstitial [34] and one peritoneal [55]. The Meddeb rise cited here is the emergency presentation after laparoscopy, not the methotrexate-era rise that preceded it [34]. Both patients already had systemic disease, or developed it soon afterwards, and untreated choriocarcinoma doubles fast enough to produce the same rise. The two reports tell the same story, and that is a fact about the map; it is not evidence that laparoscopy caused dissemination. No systematic assessment of harms was undertaken, and none is claimed.

What Should Be Reported Next

This is a reporting gap, and no trial is needed to close it. Nine items, one sentence each, would make a future report of laparoscopic or robot-assisted surgery for GTD analyzable (Table 7). None of them asks the surgeon to operate differently, and none requires information the operating team does not already hold. The request is addressed to journals and to the societies that set expectations for an operative report, not to the individual author: a case report is accepted or rejected long before anyone asks what the pneumoperitoneum pressure was.

**Table 7:** Nine items that would make a report of laparoscopic or robot-assisted surgery for GTD analyzable. The nine items are the eight technical items charted in this review plus explicit consideration of intraperitoneal spillage. Each item asks for one sentence in the operative description of a future report. GTD: gestational trophoblastic disease

Number	Item	One sentence should state
1	Uterine manipulator	Whether one was used, and if it was deliberately not used, that this was a decision
2	Proximal uterine artery control	How the uterine arteries were handled, if the uterus was in the operative field
3	Tubal occlusion	Whether the tubes were occluded, and at what point in the operation
4	Extraction route	How the specimen left the abdomen
5	Retrieval bag	Whether a bag was used
6	Morcellation	Whether the specimen was morcellated, or explicitly was not
7	Peritoneal washing cytology	Whether washings were sent, and the result
8	Pneumoperitoneum pressure	The working pressure used
9	Intraperitoneal spillage	Whether spillage was considered, and what was done about it

Until they are written, no maneuver here should become a standard because 17 reports between them describe some form of containment, or be dropped because one retrospective comparative series doubts it [59].

Limitations

The protocol was registered retrospectively, and one statement in it wrongly described how far screening had gone at that date (see Design and Registration). The searches did not cover the whole literature. Embase, CENTRAL, CNKI, and Wanfang were not searched. The last two matter most: larger series come from Chinese centers, and this review includes one of them [38]. Gynecological Surgery and Gynaecological Endoscopy are not indexed in PubMed, and both have carried eligible reports. Moreno-Collado et al. (2012) [25] is the included Gynecological Surgery report; it was reached from the reference list of Sugrue et al. (2021) [59]. Citation searching now covers 50 of the 64 included reports, so the reference lists of 14 remain unread and further eligible reports may sit in them; those 14 are the 13 Ichushi-Web-only reports for which no machine-readable full text was available, plus one report held only as an abstract. The search also missed a report in PubMed: Shen et al. (2012) never uses a laparoscopy word in its title or abstract, so the string could not find it, and it was reached by citation chasing [24].

PubMed records and citation-chased records were screened by the first author alone. The κ of 0.151 comes from the Ichushi-Web dual screen and says nothing about how reliable the PubMed screen was. One person also did all the extraction: the tallies are produced by script, and every coding rule is dated, but no second reader repeated the work. Overall, 15 of the 64 reports lack a complete full text.

Country was not coded. The third planned sensitivity analysis, on alternative denominators for retrieval bag and peritoneal washing, was not carried out because the ectopic-site flag on which those denominators depend was not locked before the tallies were formed, and recoding it afterwards would no longer be a pre-specified test. One bound can still be stated: widening the denominator to all 64 reports gives two of 64 (3.1%) for the retrieval bag and six of 64 (9.4%) for peritoneal washing cytology, against the 4.0% and 11.3% in Table 5. The two analyses that were carried out both changed the rates by less than one percentage point. The era split at 2020 is also one cut among several, and neither era is large enough to read a rate from a single item. Neither tested the denominator rules, the handling of “full text not obtained,” or which actions count as containment triggers, and the reported rates depend more on those three choices than on the two that were tested. The reports were not appraised for risk of bias. JBI guidance does not require that for a scoping review of what is reported, and no effect was estimated here [12].

## Conclusions

The laparoscopic and robot-assisted operations reported for GTD since 1995 are almost entirely single cases and appear more often outside gynecologic oncology than within it. In 29 of the 67 operations, the diagnosis was not known beforehand, so nothing about containment could have been planned. Whether or not GTD was expected, the intraoperative record is largely empty: the precautions that could contain spillage are usually not described, and where a maneuver is described, no reason is given for it. Port-site events, the one harm these guidelines name, are unreported in 43 of the 49 reports in which the field was evaluable. This review cannot say whether the laparoscopic route is safe in GTD, and neither can the literature it maps. That is not a limit of the evidence; it is a limit of what was written down. Nine details should be reported: the uterine manipulator, proximal uterine artery control, tubal occlusion, the extraction route, the retrieval bag, morcellation, peritoneal washing cytology, the pneumoperitoneum pressure, and explicit consideration of intraperitoneal spillage. If future reports state them, the next review of this literature will be able to compare what was done, instead of counting what was not recorded.

## Appendices

Appendix A: Executed search strings

No language restriction and no date restriction were applied. ETT[tiab] was omitted from the PubMed string because it retrieves endotracheal tube records. The Ichushi-Web history is reproduced as stored, after the controlled-term mapping of the term for endoscopic approach that is required to reproduce the yield of 329.

**Table 8:** Executed search strings. The PubMed query was executed as sets #1 AND #2 in a single string with no filters. The Ichushi-Web history is reproduced as stored, after the controlled-term mapping of the term for endoscopic approach that is required to reproduce the yield of 329. ETT[tiab] was omitted from the PubMed string because it retrieves endotracheal tube records. /TH and /AL are Ichushi-Web field tags for a controlled term and for all fields. Key to the Japanese terms in Ichushi-Web sets #1 and #2: 絨毛性疾患 = gestational trophoblastic disease; 胞状奇胎 = hydatidiform mole; 胞状奇胎-侵入 and 侵入奇胎 = invasive mole; 絨毛癌 = choriocarcinoma; 存続絨毛症 = persistent trophoblastic disease; 胎盤部トロホブラスト腫瘍 = placental site trophoblastic tumor; 類上皮性トロホブラスト腫瘍 = epithelioid trophoblastic tumor; 腹腔鏡 = laparoscope (the instrument term in the Ichushi-Web thesaurus); 腹腔鏡法 = laparoscopy; 腹腔鏡下 = laparoscopic; 内視鏡法 = endoscopy; 鏡視下 = endoscope-assisted; 審査腹腔鏡 = diagnostic laparoscopy; ロボット手術 = robotic surgery; ロボット支援手術 = robot-assisted surgery. Mesh: Medical Subject Headings; tiab: title/abstract field

Database and date	Set	Query exactly as executed
PubMed (National Center for Biotechnology Information web interface), 18 August 2026, no filters	#1	(“Gestational Trophoblastic Disease”[Mesh] OR “Trophoblastic Neoplasms”[Mesh] OR “Hydatidiform Mole”[Mesh] OR “Hydatidiform Mole, Invasive”[Mesh] OR “Choriocarcinoma”[Mesh] OR “Trophoblastic Tumor, Placental Site”[Mesh] OR trophoblast*[tiab] OR “hydatidiform mole”[tiab] OR “molar pregnancy”[tiab] OR “invasive mole”[tiab] OR choriocarcinoma[tiab] OR “placental site trophoblastic”[tiab] OR “epithelioid trophoblastic tumor”[tiab] OR “epithelioid trophoblastic tumour”[tiab] OR GTN[tiab] OR GTD[tiab] OR PSTT[tiab])
PubMed	#2	(“Laparoscopy”[Mesh] OR “Robotic Surgical Procedures”[Mesh] OR “Natural Orifice Endoscopic Surgery”[Mesh] OR laparoscop*[tiab] OR robot*[tiab] OR “minimally invasive”[tiab] OR pelviscop*[tiab] OR celioscop*[tiab] OR “video-assisted”[tiab] OR vNOTES[tiab] OR “natural orifice”[tiab] OR "single-port"[tiab] OR “single-site”[tiab])
PubMed	#3	#1 AND #2 (449 records)
Ichushi-Web, 20 August 2026, no year filter, conference records included	#1	絨毛性疾患/TH or 胞状奇胎/TH or 胞状奇胎-侵入/TH or 絨毛癌/TH or (絨毛性疾患/TH or 絨毛性疾患/AL) or (胞状奇胎/TH or 胞状奇胎/AL) or (胞状奇胎-侵入/TH or 侵入奇胎/AL) or (絨毛癌/TH or 絨毛癌/AL) or (存続絨毛症/TH or 存続絨毛症/AL) or (胎盤部トロホブラスト腫瘍/TH or 胎盤部トロホブラスト腫瘍/AL) or 類上皮性トロホブラスト腫瘍/AL
Ichushi-Web	#2	腹腔鏡/TH or (腹腔鏡/TH or 腹腔鏡/AL) or (腹腔鏡法/TH or 腹腔鏡下/AL) or (内視鏡法/TH or 鏡視下/AL) or 審査腹腔鏡/AL or (ロボット手術/TH or ロボット支援手術/AL) or (ロボット手術/TH or ロボット手術/AL)
Ichushi-Web	#3	#1 AND #2 (329 records)

Appendix B: Ichushi-Web conference records (supplementary layer)

**Table 9:** Twenty-four Ichushi-Web conference records forming the supplementary layer. Conference records were eligible but were not entered in the technical item table (Table 5). Titles are as screened. D&C: dilation and curettage; EMA/CO: etoposide, methotrexate and actinomycin D alternating with cyclophosphamide and vincristine; ETT: epithelioid trophoblastic tumor; LAVH: laparoscopically assisted vaginal hysterectomy; PSTT: placental site trophoblastic tumor; TLH: total laparoscopic hysterectomy

Ichushi	Year	Title (as screened)
2025173270	2025	PSTT diagnosed at laparoscopy
2025159820	2025	Presumed ectopic pregnancy; diagnostic laparoscopy showed a clinical invasive mole
2025109612	2025	Same clinical narrative as 2025159820 (possible duplicate presentation)
2024224939	2024	Presumed peritoneal pregnancy; histology: choriocarcinoma
2022193095	2021	Laparoscopic hysterectomy for invasive mole
2022192927	2021	Laparoscopic surgery for PSTT
2021128849	2020	ETT diagnosed at laparoscopy
2020191302	2019	Hydatidiform mole: D&C then TLH
2019088097	2017	LAVH diagnosing exaggerated placental site and PSTT (two cases)
2019087391	2017	Presumed ectopic pregnancy; histology: choriocarcinoma
2018356077	2018	Laparoscopic hysterectomy for invasive complete mole
2018356063	2018	Presumed ectopic pregnancy; histology: choriocarcinoma
2018208384	2016	Laparoscopic salpingectomy; histology: tubal choriocarcinoma
2017270674	2017	Laparoscopic treatment of PSTT
2015402009	2015	Presumed ectopic pregnancy; tubal partial mole
2015213025	2015	Laparoscopic hysterectomy for invasive mole
2015068771	2014	Hemoperitoneum; clinical invasive mole, laparoscopic surgery
2012014502	2006	Complete mole in which laparoscopy aided early diagnosis
2008329181	2008	Two cases: presumed ectopic pregnancy, histology: choriocarcinoma
2015068489	2014	Chemoresistant clinical invasive mole; EMA/CO plus TLH
2018208547	2016	Tubal hydatidiform mole
2019087968	2017	Ectopic complete hydatidiform mole
2019087413	2017	Left tubal pregnancy with complete mole
2021261985	2021	Diagnostic surgery for presumed ectopic pregnancy; complete mole
